# Pelvis reconstruction by proximal femur upshifting and total hip arthroplasty after radical resection of an adolescent patient pelvic Ewing's sarcoma, a case report, and literature review

**DOI:** 10.1016/j.ijscr.2023.108146

**Published:** 2023-04-12

**Authors:** Mohamed A. Mahran, Ahmed A. Khalifa, Amr El-Sayed

**Affiliations:** a Orthopaedic Department, Assiut University Hospital, Assiut, Egypt; b Orthopaedic Department, Qena Faculty of Medicine and University Hospital, South Valley University, Qena, Egypt; c Reconstructive Microsurgery Unit, Department of Orthopedics and Traumatology, Assiut University Hospitals and School of Medicine, Assiut, Egypt

**Keywords:** Pelvic reconstruction, Pelvic Ewing sarcoma, Hemipelvectomy, Total hip arthroplasty megaprostheses, Femoral upshifting

## Abstract

**Introduction and importance:**

Pelvis reconstruction after tumor resection poses a challenge, especially in younger patients where preserving the patient's function and mobility is paramount.

**Case presentation:**

A 16 years old female presented in March 2019 with vague right iliac area pain, diagnosed as pelvic Ewing's sarcoma after imaging studies (MRI and MSCT scan) and obtaining an incisional biopsy. After initial chemotherapy cycles, the tumor decreased in size, and surgical intervention in two stages was performed. The first stage was in October 2019 and consisted of pelvic resection type I and II according to Enneking and Dunham classification, proximal femur upshifting to compensate for the pelvic bone defect, and a cement spacer to fill the space of the resected proximal femur. The second stage was performed after two months and consisted of implanting a total hip arthroplasty using Megaprostheses and a cementless dual mobility acetabular cup. No local recurrence or distant metastases were detected during follow-ups. At the final follow up after 36 months, the patient showed acceptable functional outcomes (HHS score 83, and MSTS score 23 (76.7 %) points), and the radiographs showed proper implant positioning and stability.

**Clinical discussion:**

Treating pelvic Ewing's sarcoma requires a multidisciplinary team. After surgical resection, the pelvic reconstruction options include using allografts or autografts, femur upshifting, and hemipelvis prostheses, which should be chosen considering patients and tumor characteristics as well as surgical team efficiency.

**Conclusion:**

Reconstructing the pelvic defect after bone tumor resection by proximal femoral upshifting is a valid biological option with acceptable outcomes.

## Highlights

• Pelvic reconstruction after bone tumor resection could be challenging
• Various options for reconstruction, including biological and non-biological options
• We utilized femoral upshifting combined with total hip arthroplasty for pelvic reconstruction post Ewing sarcoma resection.
• Femoral upshifting with total hip arthroplasty provided acceptable functional outcomes and patient satisfaction.

## 1 Introduction

Reconstructing the pelvic ring after pelvic bone tumor resection is challenging, especially at a younger age, where the reconstruction procedure aims to restore patient function and mobility [1], [2]. Furthermore, if the tumor involves the acetabulum, involvement of the hip joint in the reconstruction procedure is paramount [1], [3].

Although some authors reported accepted results after leaving the hip to fail [4], [5]; however, various techniques for hip joint reconstruction were proposed, such as ipsilateral proximal femoral upshifting, modular hemipelvis prosthesis, biological reconstruction (autologous non-vascularized fibular graft, autologous iliac crest bone graft) pelvic allografts, and allograft-prosthetic reconstructions [1], [6], [7], [8], [9].

Puget and Utheza, in 1986, were the first to describe the technique of ipsilateral femur upshifting to reconstruct the resected ipsilateral pelvic bone by plate and screws fixation, then a cemented cup was inserted on the recreated acetabular side, and the femur was replaced by a Megaprostheses total hip arthroplasty (THA) [7], [10]. This technique provided a biological reconstruction option that avoids the fractures, failures, and lack of incorporation occurring with allografts transplants [6].

In this report, we describe the results of managing a relatively young patient diagnosed with pelvic Ewing's sarcoma (ES) treated by femur upshifting and a Megaprostheses THA with dual mobility acetabular cup.

## 2 Presentation of case

**Fig. 1:**
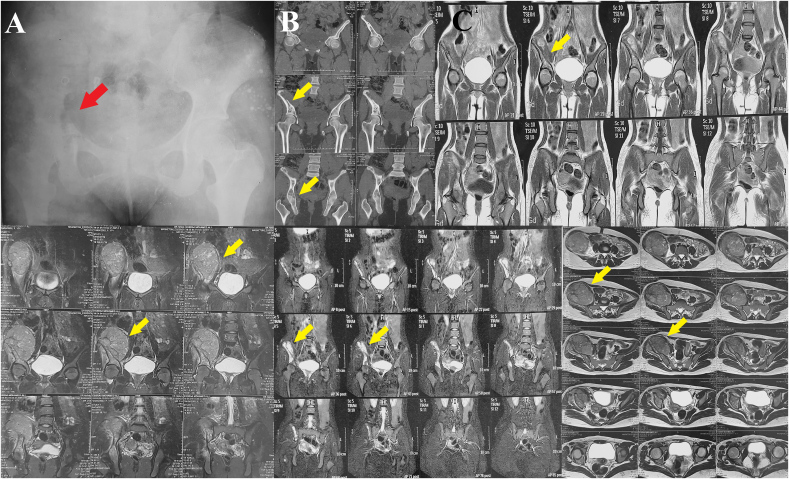
Preoperative imaging studies. A: pelvis anteroposterior plain radiographic view showing erosion of the inner aspect of the right acetabulum (red arrow). B: a CT scan of the pelvis showing affection of the right iliac bone (yellow arrow). C: Different pelvis MRI cuts showing a soft tissue mass arising within the iliacus muscle in the medial to the right iliac bone (yellow arrows). (For interpretation of the references to colour in this figure legend, the reader is referred to the web version of this article.)

In line with the SCARE criteria [11], we report a case of a female patient 16 years old presented in March 2019 to our OPD with her parents complaining of progressive right iliac area pain radiating to her right hip joint over the past two months, not responding to analgesics; she also noted a feeling of fullness proximal to her right hip and a slight limp while walking. Clinical evaluation revealed normal skin over the right hip and iliac region, and no swelling was felt; however, her hip joint motion had painful and limited last 15 degrees of internal rotation. The distal neurovascular bundle was intact. An initial imaging study in the form of a plan radiograph anteroposterior view of the pelvis showed discontinuity and a destructive lesion on the inner side of the right acetabulum. Further imaging studies in the form of a Multi-slice CT scan (MSCT) and MRI of the pelvis revealed a mass in the iliacus muscles 5*7.5 cm in diameter with the erosion of the inner table of the iliac bone, giving features of a malignant lesion (Fig. 1, Fig. S1). Further evaluation in the form of a whole-body MRI revealed no other lesions.

An open incisional biopsy was performed in April 2019, which revealed Ewing's sarcoma. After a discussion with the oncological medicine team, they decided that the patient should undergo chemotherapy before any surgical intervention [12]. After the initial chemotherapy sessions (six cycles, 12 weeks) were completed, the MSCT and MRI scans were repeated in July and September 2019, which revealed a reduction in the lesion size to 3.3*6.2 cm (Fig. S2). Chemotherapy was held for three weeks. The decision was made to treat the patient surgically in two stages.

**Fig. 2:**
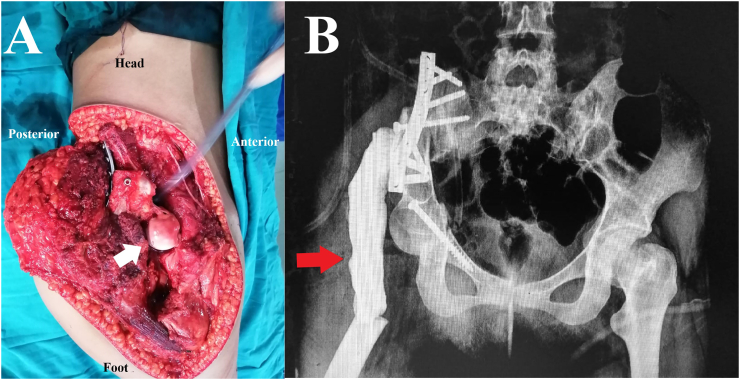
Surgical intervention, the first stage formed of, A: resection Type I and II according to the classification by Enneking and Dunham of the right hemipelvis and upshifting of the proximal femur to reconstruct the defect (white arrow pointing to the femoral head placed near the resected native acetabulum). B: immediate postoperative pelvis anteroposterior plain radiographic view showing the proximal femur upshifted and fixed to the pelvis and a cement spacer (red arrow) placed to compensate for the resected proximal femoral segment. (For interpretation of the references to colour in this figure legend, the reader is referred to the web version of this article.)

The first stage was performed in October 2019 through an extended iliofemoral approach starting at the level of the sacroiliac joint superiorly and posteriorly, then winding down over the iliac crest to continue over the lateral aspect of the thigh in line with the direct lateral approach of the hip with an incision centered over the greater trochanter. This stage consisted of right hemipelvectomy (resection Type I and II according to the classification by Enneking and Dunham [3]), upshifting of the right proximal femur (as a vascularized based on the medial femoral circumflex artery), where the femur was osteotomized below the level of the lesser trochanter by 2 cm and transferred proximally in an upside down fashion (the femoral head facing the obturator foramen and the femoral shaft toward the sacroiliac joint) and was internally fixed in place using plates and screws, and a cement spacer was placed compensating for the resected proximal femur and to maintain alignment and soft tissue tension (Fig. 2, Fig. S3). Postoperatively, the patient spent two weeks in the hospital, which was uneventful, during which the patient was kept non-weight bearing on the operated side, and sutures were removed just before discharge with no wound healing problems. The whole specimen was sent for histopathological evaluation, which confirmed the diagnosis of Ewing's sarcoma, and the response to chemotherapy was grade I according to the scoring system proposed by Picci et al. [13]; they also reported that the acetabular and iliac bony margins were free of infiltration (Fig. S4).

**Fig. 3:**
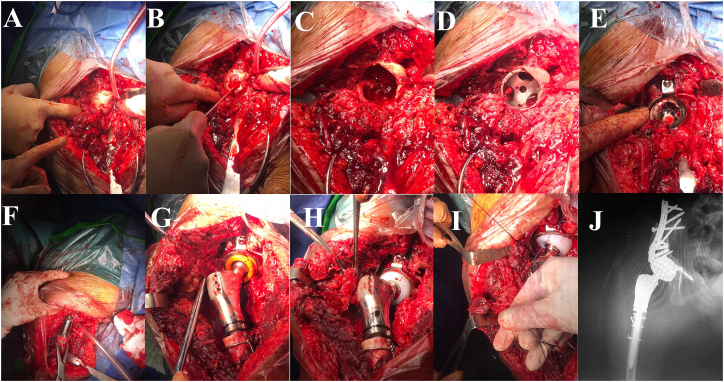
Surgical intervention, the second stage formed of, A: approaching the pelvis and exposing the femoral head. B: preliminary identification of the femoral head center to start proper reaming for creating an acetabular cavity. C: after acetabular reaming completion and the creation of a new acetabulum. D: acetabular cup trial in place. E: the actual cementless acetabular dual mobility cup was placed, and a supraacetabular screw was used for fixation. F: femoral shaft preparation for insertion of a proximal femoral replacement prosthesis. G: after insertion of the trial component. H: the actual final component is in place and tested for stability. I: soft tissue repair and reattachment to the holes in the proximal segment of the femoral component. J: immediate postoperative anteroposterior view of the right hip showing proper positioning of the implants.

**Fig. 4:**
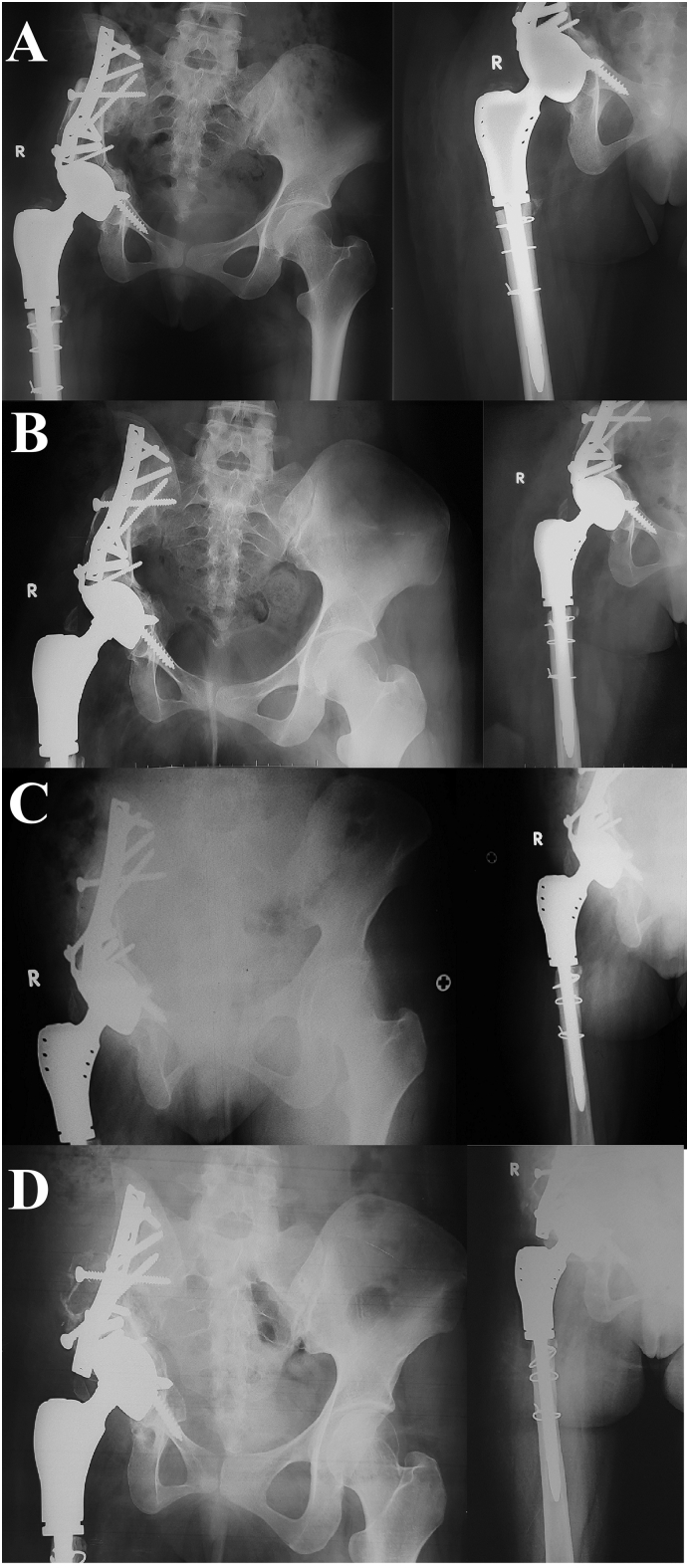
Postoperative and follow up radiographic evaluation (till the last follow up) showing proper positioning and stability of the implants. A: first postoperative follow up visit (January 2020). B: Follow up in June 2020. C: June 2021. D: last follow up in November 2022.

The second stage was performed in December 2019 and consisted of Megaprostheses THA using a cementless dual mobility acetabular cup (providing more stability to compensate for the deficient hip abductors) and a cementless tumor prosthesis femoral stem, and reattachment of the remaining abductor muscles to the proximal segment of the femoral stem (Fig. 3). Postoperatively, after confirmation of the proper positioning of the implants, the patient was instructed to toe-touch weight bearing; at two weeks, the sutures were removed, and the wound healed without complications. The oncology team recommended continuation of chemotherapy (for further four cycles) starting one month postoperatively. The subsequent follow-ups (every three months for the first two years consisting of clinical examination, radiographs, and chest CT scan, then every six months after that) of the patient were uneventful, no local recurrence or distant metastases were detected, and the implants showed preserved position with no signs of loosening (Fig. 4). At the last follow up at 36 months postoperatively, the patient was walking without support with a moderate limp, she could sit cross-legged, and her hip functional outcome was good according to Harris Hip Score HHS (score 83) and scored 23 (76.7 %) points (out of a total 30 points) according to Musculoskeletal Tumor Society (MSTS) score. Assessment of the patient's life quality was performed according to the SF-12 questionnaire, which revealed a physical score (PCS-12) of 46.6 and a mental score (MCS-12) of 51.3.

## 3 Discussion

Ewing's sarcoma is considered the second most common tumor in pediatrics and the fourth comments among all tumors, occurring commonly during the life second decade at an age ranging from 13 to 19 years at an incidence of 1.2–2.93 in one million population and its incidence of occurrence in the pelvis reaches up to 20 % [2], [14], [15], [16]. The 5-year survival after ES was shown to be better in a patient treated with surgical resection accompanied with chemotherapy than in patients who only received chemo or radiotherapy [17].

In dealing with malignant bone lesions, the surgeon should aim for radical en bloc surgical resection of the lesion with tumor-free margins, followed by the challenging task of anatomical reconstruction to preserve function, especially in younger patients; furthermore, in case of pelvic lesions, these steps are more challenging, and the reconstructive options are technically and financially demanding [1], [8], [9], [10].

For resection type II, which involves removing the periacetabular region, hip reconstruction is mandatory in adults for restoring the hip joint function; however, in skeletally immature patients with open triradiate cartilage, this physic could act as a barrier against tumor extension, so some surgeons consider this area as the oncological margin for tumor resection with further preservation of the acetabulum [18], [19]. However, in the current case, the patient's radiographs showed a closed triradiate cartilage by the time of presentation, so we managed the patient as an adult and performed type I and II resections with further reconstructing hip by ipsilateral femur upshifting and a THA Megaprostheses. Furthermore, to avoid the mechanical failures reported in previous studies (mainly dislocation), we used a dual mobility acetabular component to compensate for the deficient hip abductors.

Puget and Utheza originally described performing this technique in three patients (2 had pelvic metastases, and one had Plasmacytoma); the youngest was 45 years old with an acceptable outcome [7], [10].

Biau et al. in 2009 included 13 patients having a median age of 51 years (the youngest patient was 22) diagnosed with pelvic malignant lesions; most of them were chondrosarcomas; they treated their patients by tumor resection and reconstructing the pelvis by uplifting the proximal femur followed by a cemented THA, they described inserting the femoral autograft where the femoral head rests against the iliac bone and the shaft is resting on the pubic rami, after a median follow up of 49 months, seven patients were alive and reported acceptable functional outcomes; however, they reported that four patients underwent revision, 1 for aseptic loosening and 2 for infection [20].

Laffosse et al. 2012, reported on ten patients having a mean age of 38 years (the youngest was 19 years old), including three patients diagnosed with Ewing sarcomas, on a median of 40 months follow up, the authors reported that the femoral graft was completely integrated in five patients and partially in three, and no cases of fracture or non-union. By a median follow up of 82 months, five patients were living and reported acceptable functional outcomes; all were walking without support. The authors mentioned that five patients required nine revisions, seven of which were attributed to tumor recurrence [21].

Lin et al., in 2018, retrospectively reviewed 11 patients (the youngest was 19 years old) diagnosed with pelvic tumors (Type II resections as defined by Enneking and Dunham); 7 had chondrosarcomas, 3 Ewing sarcomas, and 2 GCT, all were treated using the technique of femur upshifting, three patients died after tumor recurrence and one lost during follow up; however, the authors reported that the remaining seven patients were free of recurrence after a mean follow up of 37 months, and reported a median MSTS function score of70%. They reported a high rate of complications where two patients had mechanical implant failure, one non-union, and two infections [1].

The concept of using the proximal femoral segment as an autograft for reconstruction is justifiable by many facts. First, the femoral autograft is present within the same surgical field. Second, it could provide an appropriate amount, quality, and shape of bone autograft as the femoral head and neck with the trochanteric area constitute a robust cortico-cancellous graft having a curved shape and could be fashioned as an acetabular cavity. Third, this robust biological reconstruction allows early weight bearing and avoids complications of allografts reconstruction [1], [6], [10].

In a cadaveric anatomical study by Wang et al. evaluating the suitability of the proximal femur for reconstructing the pelvic bony defect after resecting pelvic lesions, the authors studied 13 Chinese males fresh-frozen Chinese cadavers; after evaluating various anatomical measurements, the authors concluded that the proximal femoral autograft is a suitable and biologic option for reconstruction on the condition that proper preoperative planning and evaluation of the size be resected and the needed for reconstruction [22].

The technique is also prone to some limitations, first is the high technical demands required to perform this procedure, as some authors reported that complications occurred in the early cases at the start of the technique learning curve [20]. Second, most studies reported a high rate of complications, apart from tumor recurrence, non-union, infection, and mechanical failures [1], [21]. Third, the technique only fits some types of resections, as if the resection extends to zone I, some authors suggested that there will not be adequate stability with hip abductor dysfunction as the abductor muscles are already partially removed with the resection [20]. Lastly, the upshifted femur could offer limited bone diameter with subsequent use of a small acetabular cup which predisposes to early failures, as shown in the study by Traub et al., where the authors reported that failures were associated with the smallest cup diameters (40 mm) [20].

## 4 Conclusion

Although technically demanding, femur upshifting is an appealing, thriving, and biological technique to reconstruct the pelvic bone defect after Ewing's sarcoma resection in a relatively young patient. The implantation of a Megaprostheses THA and a dual mobility acetabular cup restored hip function and stability.

## Ethical approval

This case report was carried out following the declarations of Helsinki and did not contain any experimental studies with human participants or animals performed by any of the authors. The ethical committee of our institution waived the ethical approval, as this was considered part of the usual patient care.

## Funding

N/A

## Author contribution

M.A.M and A.E. conceived the case report and performed the surgeries. A.A.K. carried out data acquisition and assessment. A.A.K. and M.A.M. performed a literature search, drafted the manuscript, and designed the figures; A.E. did the critical revision. All authors discussed the results and commented on the manuscript. All authors read and approved the final manuscript. The first and the second authors contributed equally to the manuscript.

## Guarantor

M.A.M.

## Provenance and peer review

Not commissioned, externally peer reviewed.

## Consent

Written informed consent was obtained from the patient's parents/legal guardian for publication of this case report and accompanying images. A copy of the written consent is available for review by the Editor-in-Chief of this journal on request.

## Conflict of interest statement

N/A

## Appendix A Supplementary data

Supplementary figures Image 1

## References

[1] Lin N., Li H., Li W., Huang X., Liu M., Yan X. (2018). Upshifting the ipsilateral proximal femur may provide satisfactory reconstruction of periacetabular pelvic bone defects after tumor resection. Clin. Orthop. Relat. Res..

[2] Fan H., Guo Z., Fu J., Li X., Li J., Wang Z. (2017). Surgical management of pelvic Ewing's sarcoma in children and adolescents. Oncol. Lett..

[3] Enneking W.F., Dunham W.K. (1978). Resection and reconstruction for primary neoplasms involving the innominate bone. J. Bone Joint Surg. Am..

[4] Hoffmann C., Gosheger G., Gebert C., Jurgens H., Winkelmann W. (2006). Functional results and quality of life after treatment of pelvic sarcomas involving the acetabulum. J. Bone Joint Surg. Am..

[5] Schwartz A.J., Kiatisevi P., Eilber F.C., Eilber F.R., Eckardt J.J. (2009). The friedman-eilber resection arthroplasty of the pelvis. Clin. Orthop. Relat. Res..

[6] Traub F., Andreou D., Niethard M., Tiedke C., Werner M., Tunn P.U. (2013). Biological reconstruction following the resection of malignant bone tumors of the pelvis. Sarcoma.

[7] Puget J., Utheza G. (1986). Reconstruction of the iliac bone using the homolateral femur after resection for pelvic tumor. Rev Chir Orthop Reparatrice Appar Mot..

[8] Angelini A., Calabro T., Pala E., Trovarelli G., Maraldi M., Ruggieri P. (2015). Resection and reconstruction of pelvic bone tumors. Orthopedics.

[9] Dominkus M., Darwish E., Funovics P. (2009). Reconstruction of the pelvis after resection of malignant bone tumours in children and adolescents. Recent Results Cancer Res..

[10] Puget J., Utheza G. (2014). Reconstruction of the iliac bone using the homolateral femur after resection for pelvic tumor. Orthop. Traumatol. Surg. Res..

[11] Agha R.A., Franchi T., Sohrabi C., Mathew G., Kerwan A., Group S. (2020). The SCARE 2020 guideline: updating consensus Surgical CAse REport (SCARE) guidelines. Int J Surg..

[12] Grier H.E., Krailo M.D., Tarbell N.J., Link M.P., Fryer C.J., Pritchard D.J. (2003). Addition of ifosfamide and etoposide to standard chemotherapy for Ewing's sarcoma and primitive neuroectodermal tumor of bone. N. Engl. J. Med..

[13] Picci P., Bohling T., Bacci G., Ferrari S., Sangiorgi L., Mercuri M. (1997). Chemotherapy-induced tumor necrosis as a prognostic factor in localized Ewing's sarcoma of the extremities. J. Clin. Oncol..

[14] Abed R., Grimer R. (2010). Surgical modalities in the treatment of bone sarcoma in children. Cancer Treat. Rev..

[15] Frassica F.J., Frassica D.A., Pritchard D.J., Schomberg P.J., Wold L.E., Sim F.H. (1993). Ewing sarcoma of the pelvis. Clinicopathological features and treatment. J. Bone Joint Surg. Am..

[16] Whelan J., McTiernan A., Cooper N., Wong Y.K., Francis M., Vernon S. (2012). Incidence and survival of malignant bone sarcomas in England 1979–2007. Int. J. Cancer.

[17] Sucato D.J., Rougraff B., McGrath B.E., Sizinski J., Davis M., Papandonatos G. (2000). Ewing's sarcoma of the pelvis. Long-term survival and functional outcome. Clin. Orthop. Relat. Res..

[18] Cheung W.H., Lee K.M., Fung K.P., Leung K.S. (2001). Growth plate chondrocytes inhibit neo-angiogenesis – a possible mechanism for tumor control. Cancer Lett..

[19] Sales de Gauzy J., Lafontan V., Ursei M., Accadbled F. (2014). Ewing sarcoma of the acetabulum in children: a "growth plate-based" surgical strategy. J. Pediatr. Orthop..

[20] Biau D.J., Thevenin F., Dumaine V., Babinet A., Tomeno B., Anract P. (2009). Ipsilateral femoral autograft reconstruction after resection of a pelvic tumor. J. Bone Joint Surg. Am..

[21] Laffosse J.M., Pourcel A., Reina N., Tricoire J.L., Bonnevialle P., Chiron P. (2012). Primary tumor of the periacetabular region: resection and reconstruction using a segmental ipsilateral femur autograft. Orthop Traumatol Surg Res..

[22] Wang S., Xiong J., Zhan C., Wang A., Chen Y., Jiang Q. (2012). The anatomy of proximal femoral autografts for pelvic reconstruction: a cadaveric study. Surg. Radiol. Anat..

