# Supplementary material for: Pelvis reconstruction by proximal femur upshifting and total hip arthroplasty after radical resection of an adolescent patient pelvic Ewing's sarcoma, a case report, and literature review
Source: Int J Surg Case Rep. 2023 Apr 12;106:108146. doi: 10.1016/j.ijscr.2023.108146 (PMC10130465; doi:10.1016/j.ijscr.2023.108146)
Supplement: Supplementary file 1 — Supplementary figures [file mmc1.pdf]

Figure S1: Pre-Chemotherapy CT scan and MRI images showing the magnitude and the extent of the tumor mass affecting the right iliac fossa.

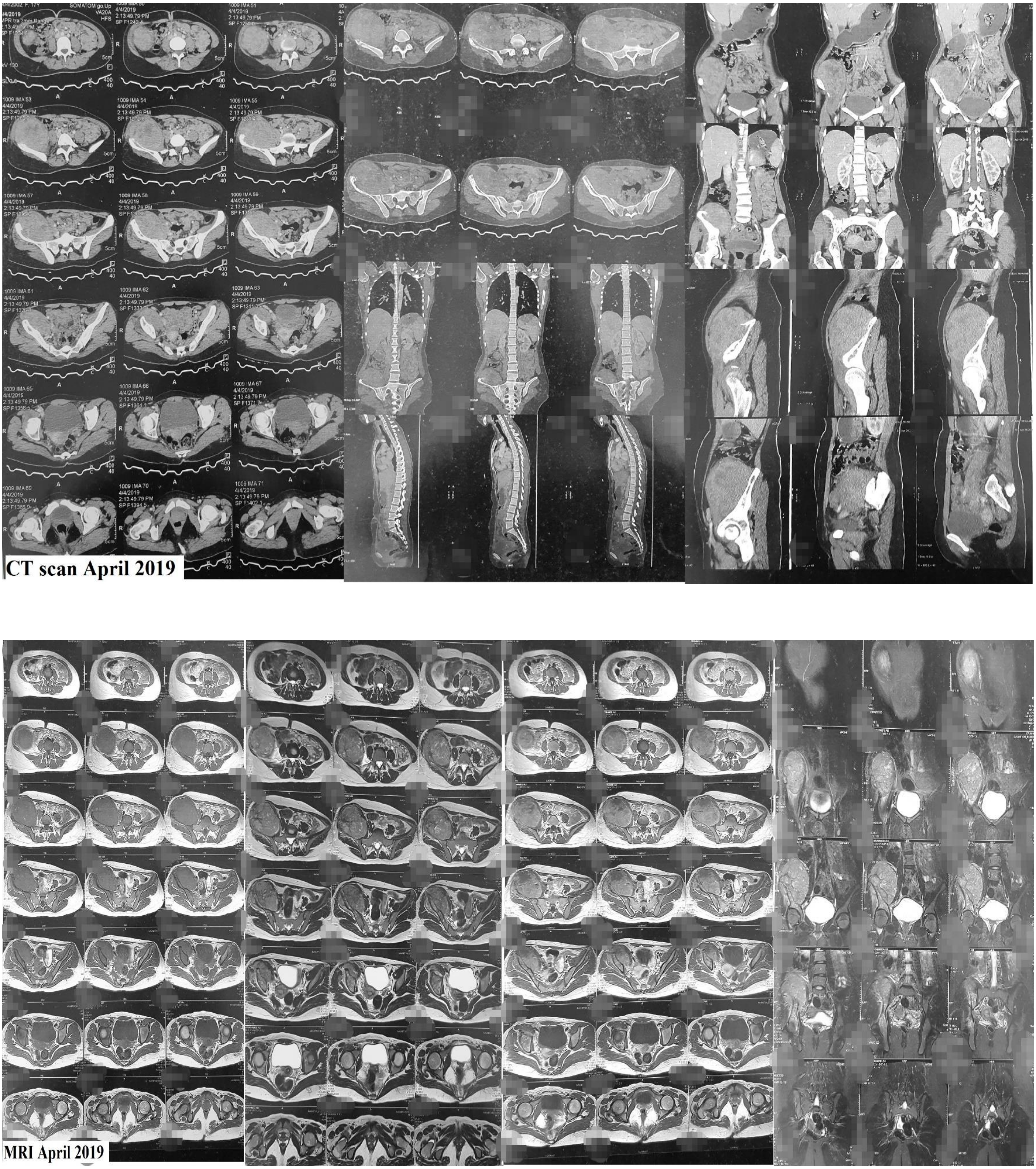

Figure S2: Post-chemotherapy and preoperative CT scans and MRI images showing tumor size reduction.

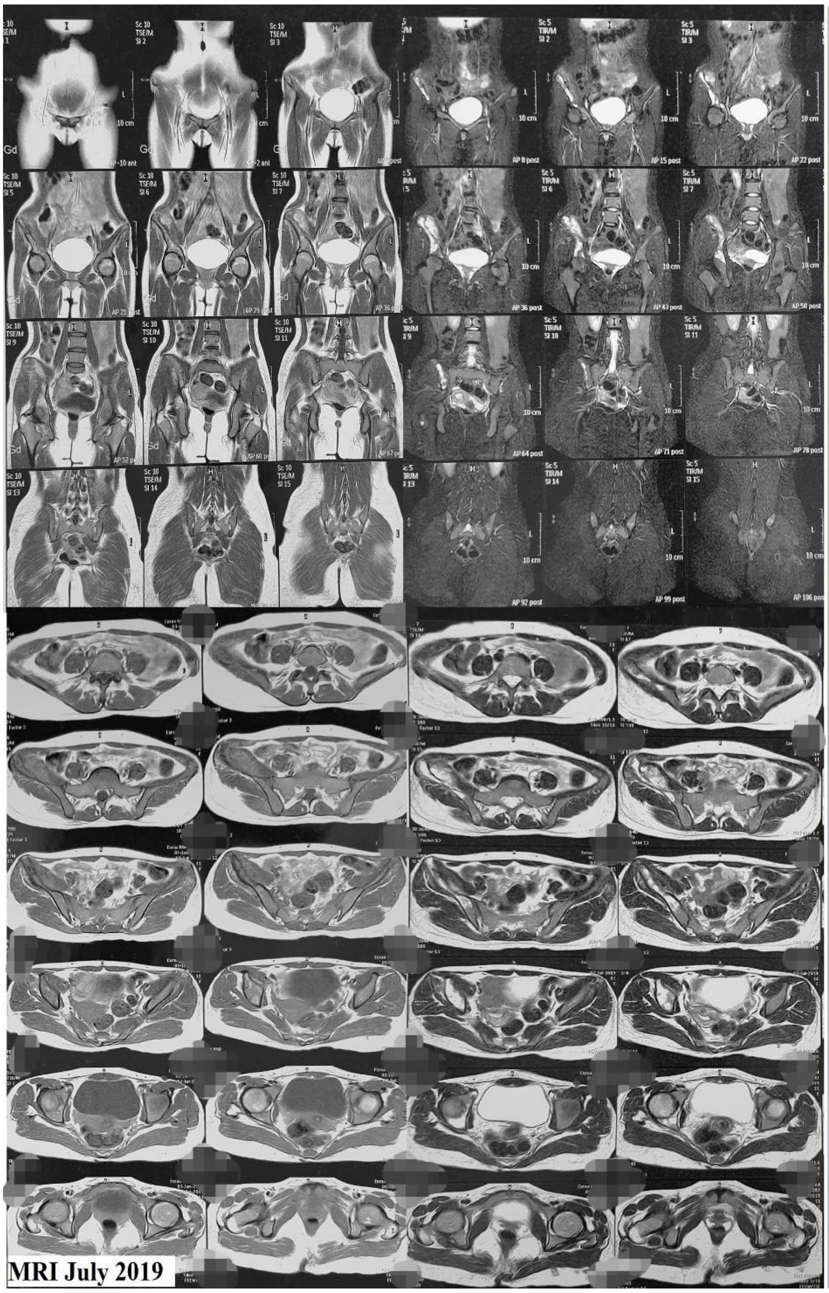

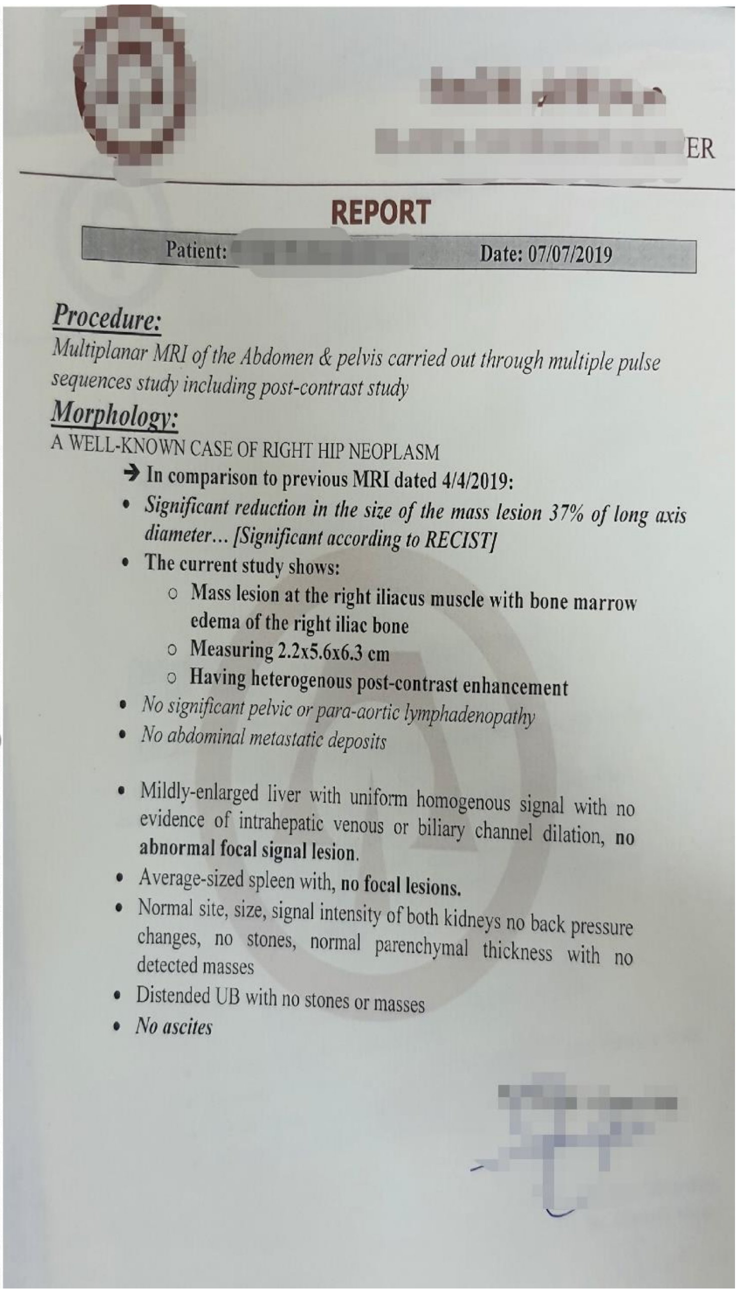
[redacted]

[redacted]ER

REPORT

Patient: [redacted] **Date: 07/07/2019**

Procedure:

Multiplanar MRI of the Abdomen & pelvis carried out through multiple pulse sequences study including post-contrast study

Morphology:

A WELL-KNOWN CASE OF RIGHT HIP NEOPLASM

➔ **In comparison to previous MRI dated 4/4/2019:**

- ***Significant reduction in the size of the mass lesion 37% of long axis diameter… [Significant according to RECIST]***
- **The current study shows:**
  - **Mass lesion at the right iliacus muscle with bone marrow edema of the right iliac bone**
  - **Measuring 2.2x5.6x6.3 cm**
  - **Having heterogenous post-contrast enhancement**
- *No significant pelvic or para-aortic lymphadenopathy*
- *No abdominal metastatic deposits*

- Mildly-enlarged liver with uniform homogenous signal with no evidence of intrahepatic venous or biliary channel dilation, **no abnormal focal signal lesion.**
- Average-sized spleen with, **no focal lesions.**
- Normal site, size, signal intensity of both kidneys no back pressure changes, no stones, normal parenchymal thickness with no detected masses
- Distended UB with no stones or masses
- *No ascites*

[redacted]

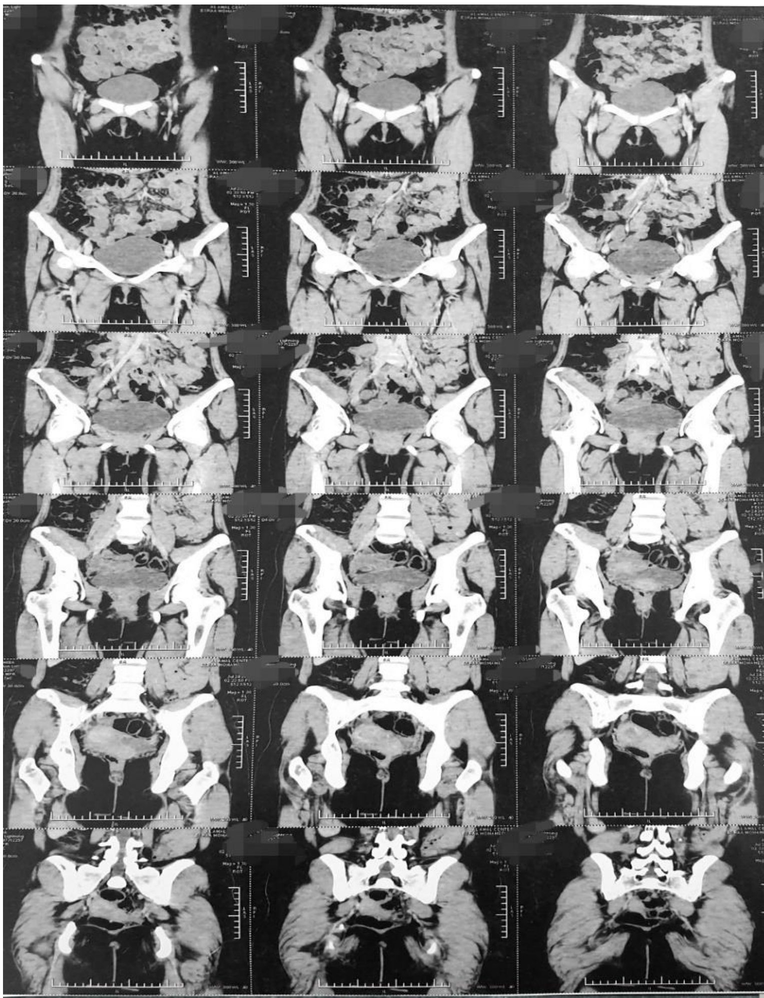

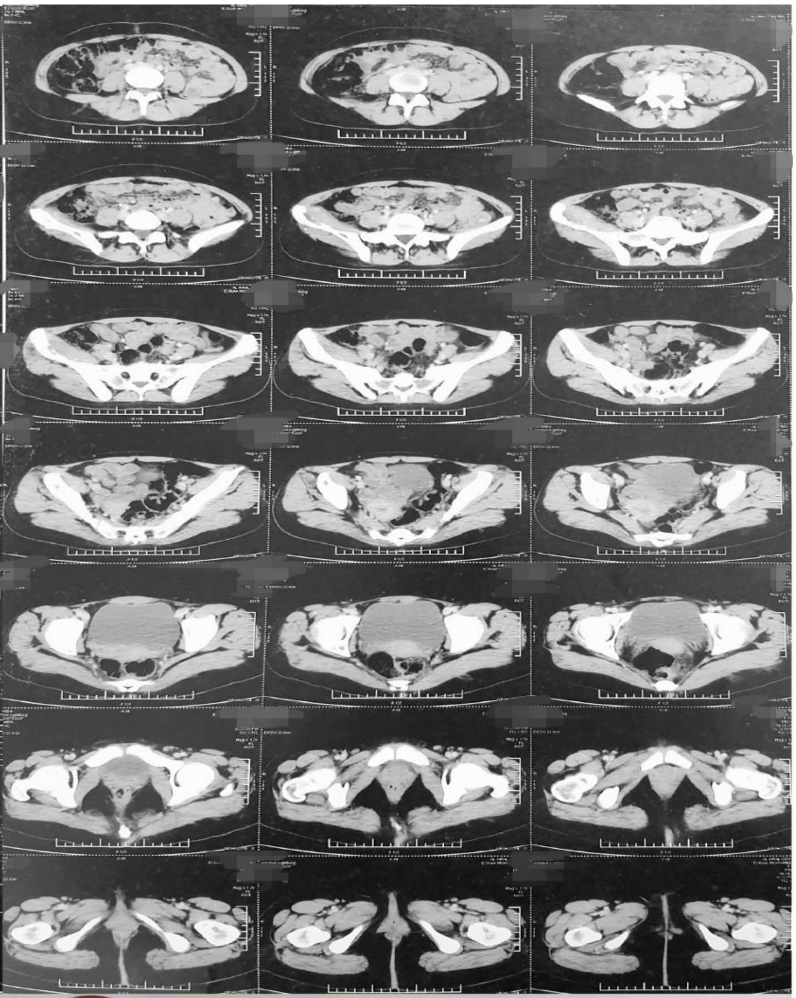

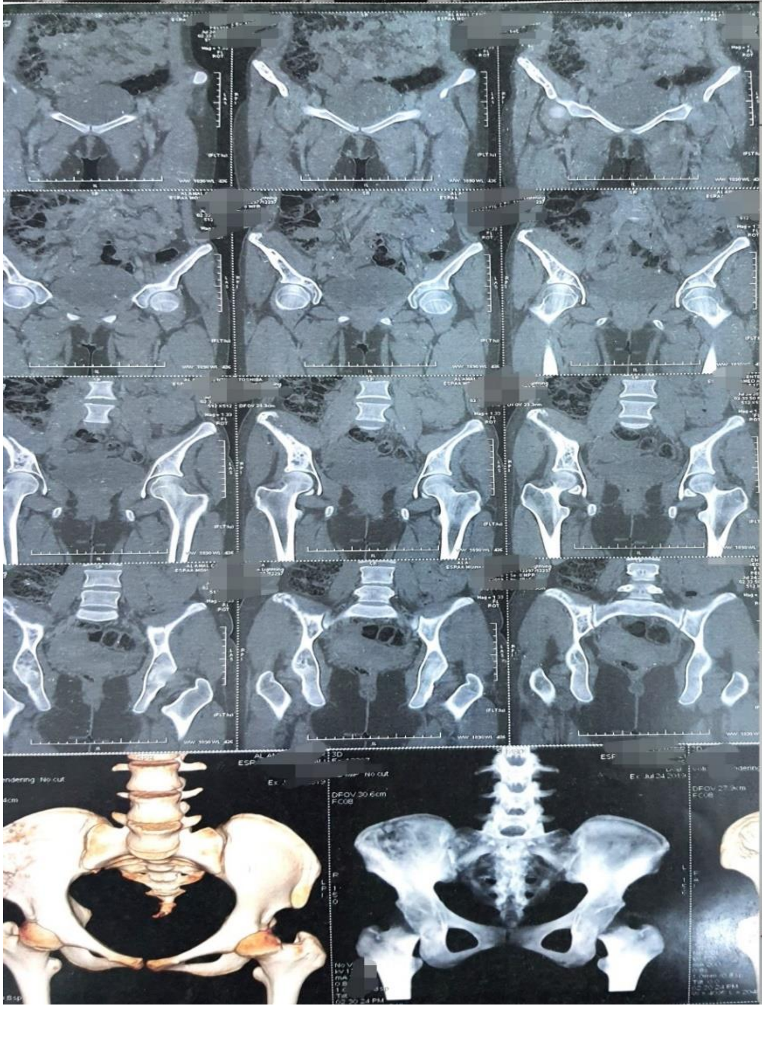

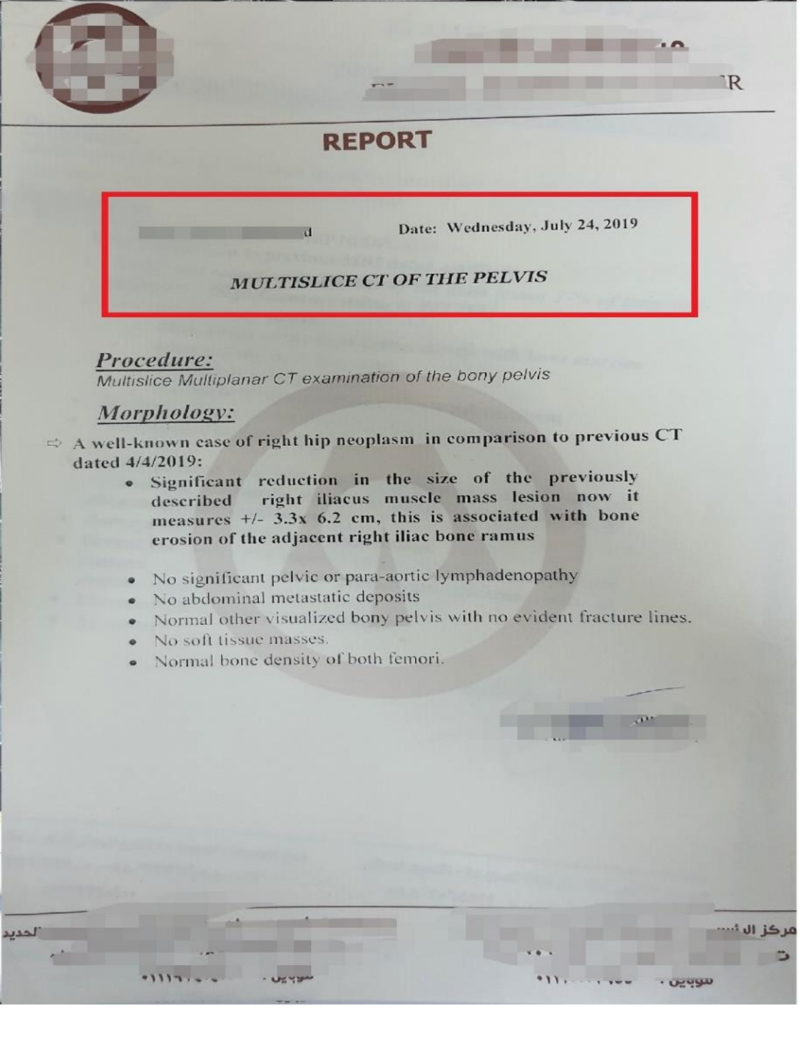
[redacted]

REPORT

[redacted] Date: Wednesday, July 24, 2019

MULTISLICE CT OF THE PELVIS

Procedure:
Multislice Multiplanar CT examination of the bony pelvis

Morphology:

⇨ A well-known case of right hip neoplasm in comparison to previous CT dated 4/4/2019:

- Significant reduction in the size of the previously described right iliacus muscle mass lesion now it measures +/- 3.3x 6.2 cm, this is associated with bone erosion of the adjacent right iliac bone ramus

- No significant pelvic or para-aortic lymphadenopathy
- No abdominal metastatic deposits
- Normal other visualized bony pelvis with no evident fracture lines.
- No soft tissue masses.
- Normal bone density of both femori.

[redacted]

[redacted]

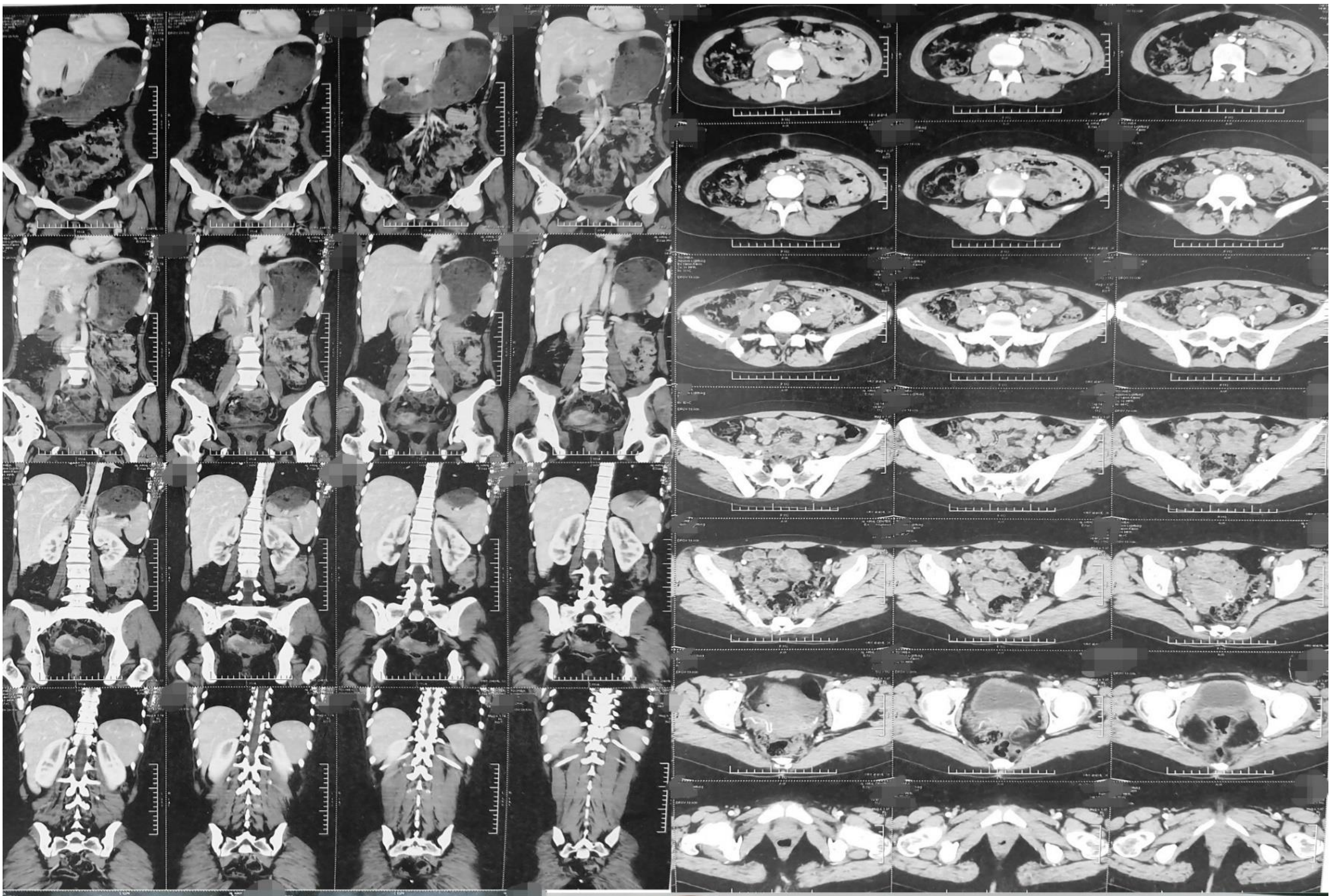

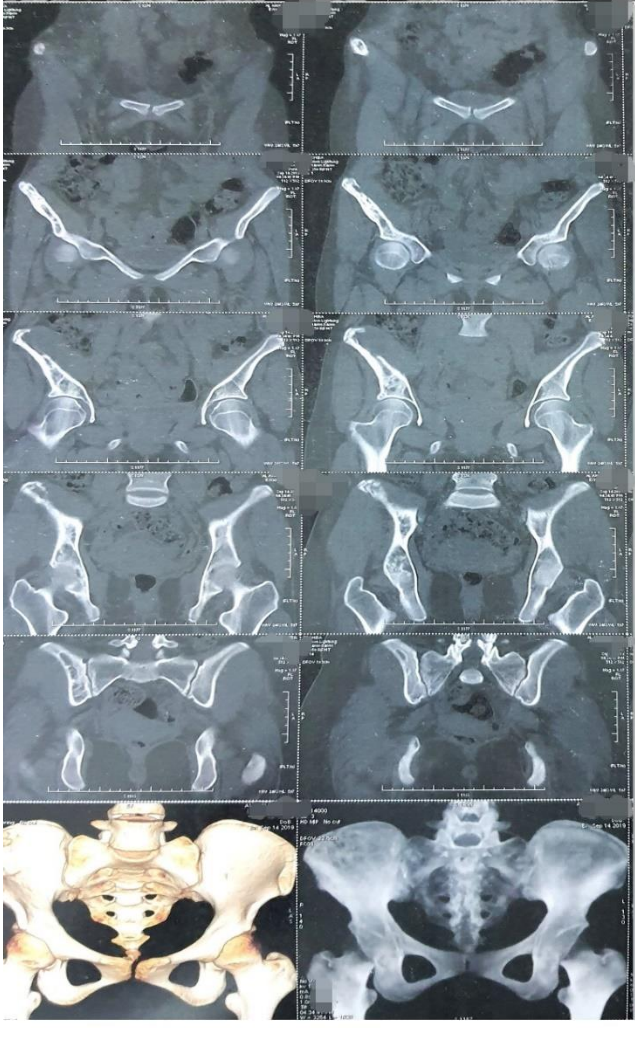

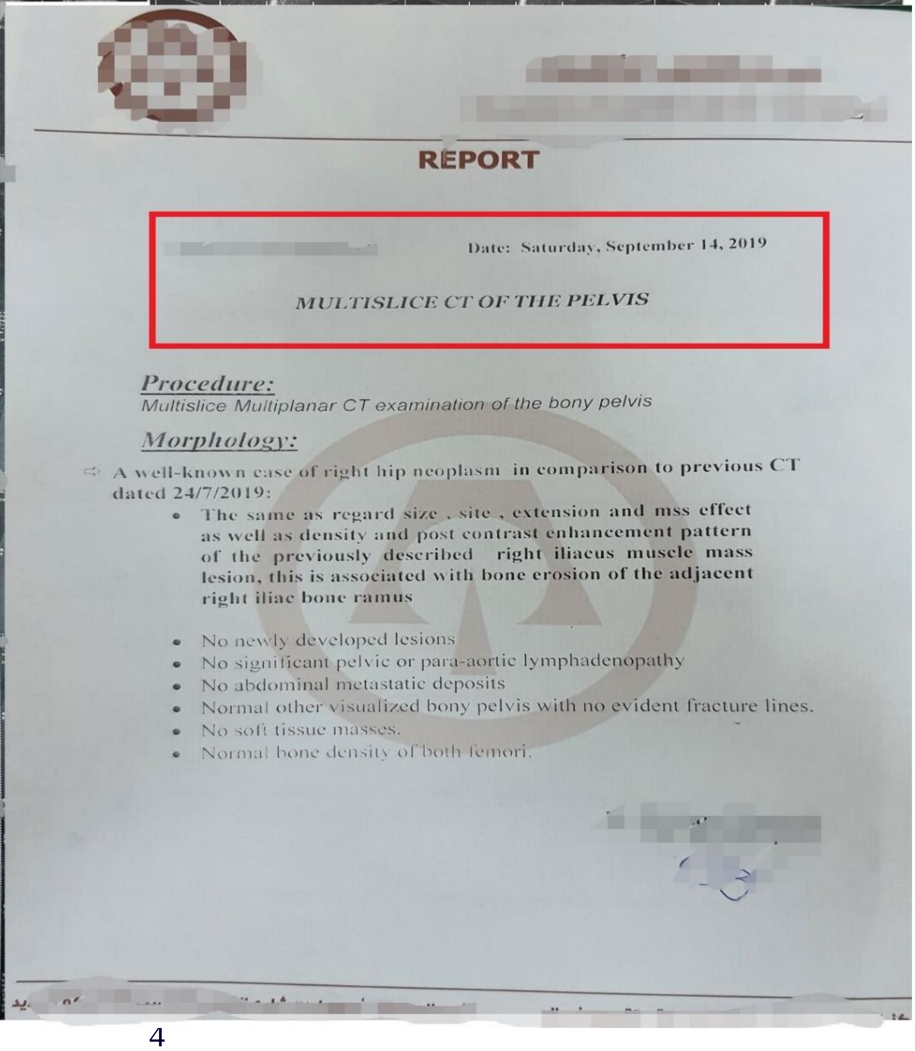

REPORT

Date: Saturday, September 14, 2019

MULTISLICE CT OF THE PELVIS

Procedure:

Multislice Multiplanar CT examination of the bony pelvis

Morphology:

⇨ A well-known case of right hip neoplasm in comparison to previous CT dated 24/7/2019:

- The same as regard size , site , extension and mss effect as well as density and post contrast enhancement pattern of the previously described right iliacus muscle mass lesion, this is associated with bone erosion of the adjacent right iliac bone ramus

- No newly developed lesions
- No significant pelvic or para-aortic lymphadenopathy
- No abdominal metastatic deposits
- Normal other visualized bony pelvis with no evident fracture lines.
- No soft tissue masses.
- Normal bone density of both femori.

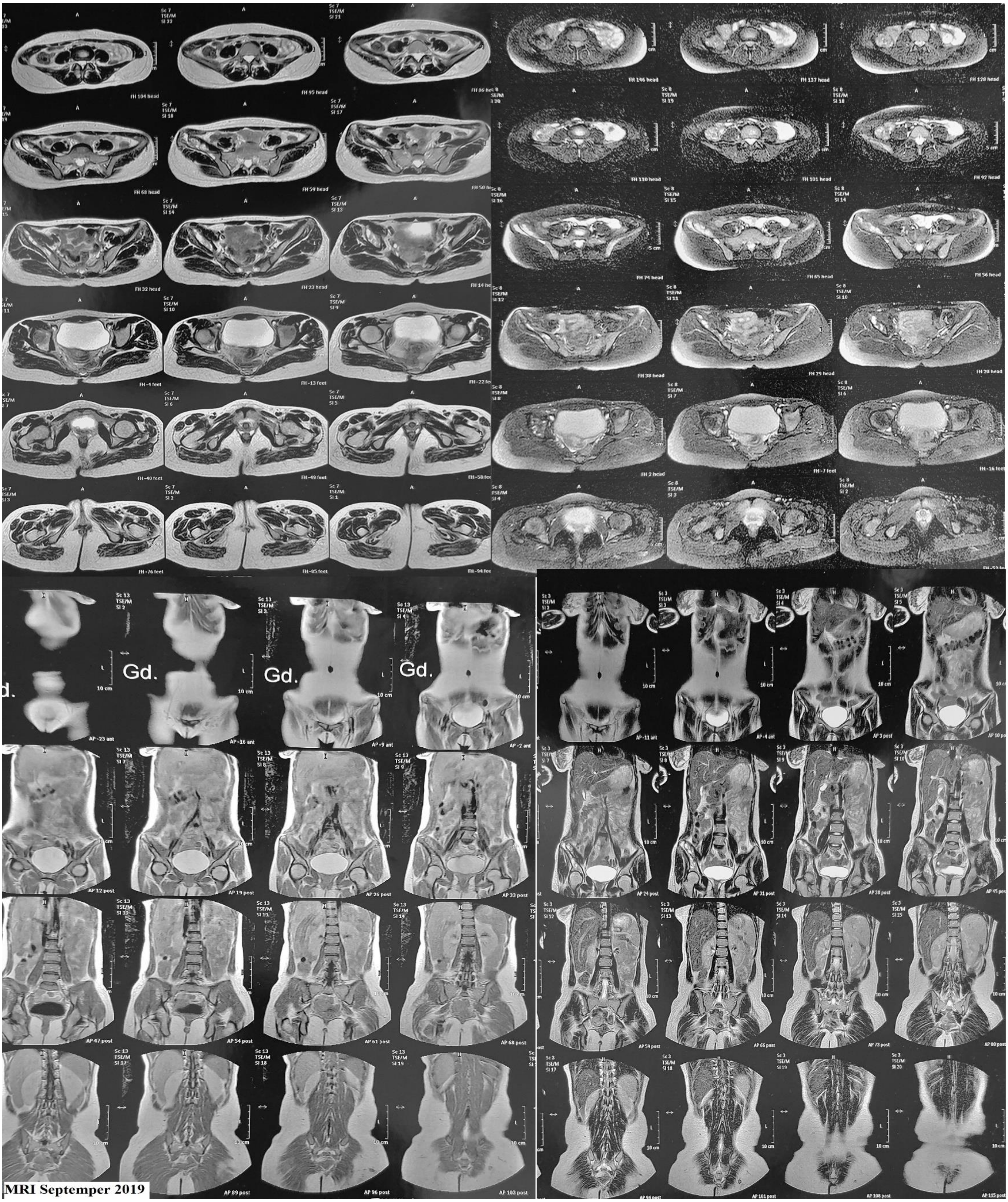
Gd.
Gd.
Gd.
MRI Septemper 2019

Figure S3: Post-first stage CT scan showing the stability of the upshifted femur autograft, and no evidence of tumor recurrence.

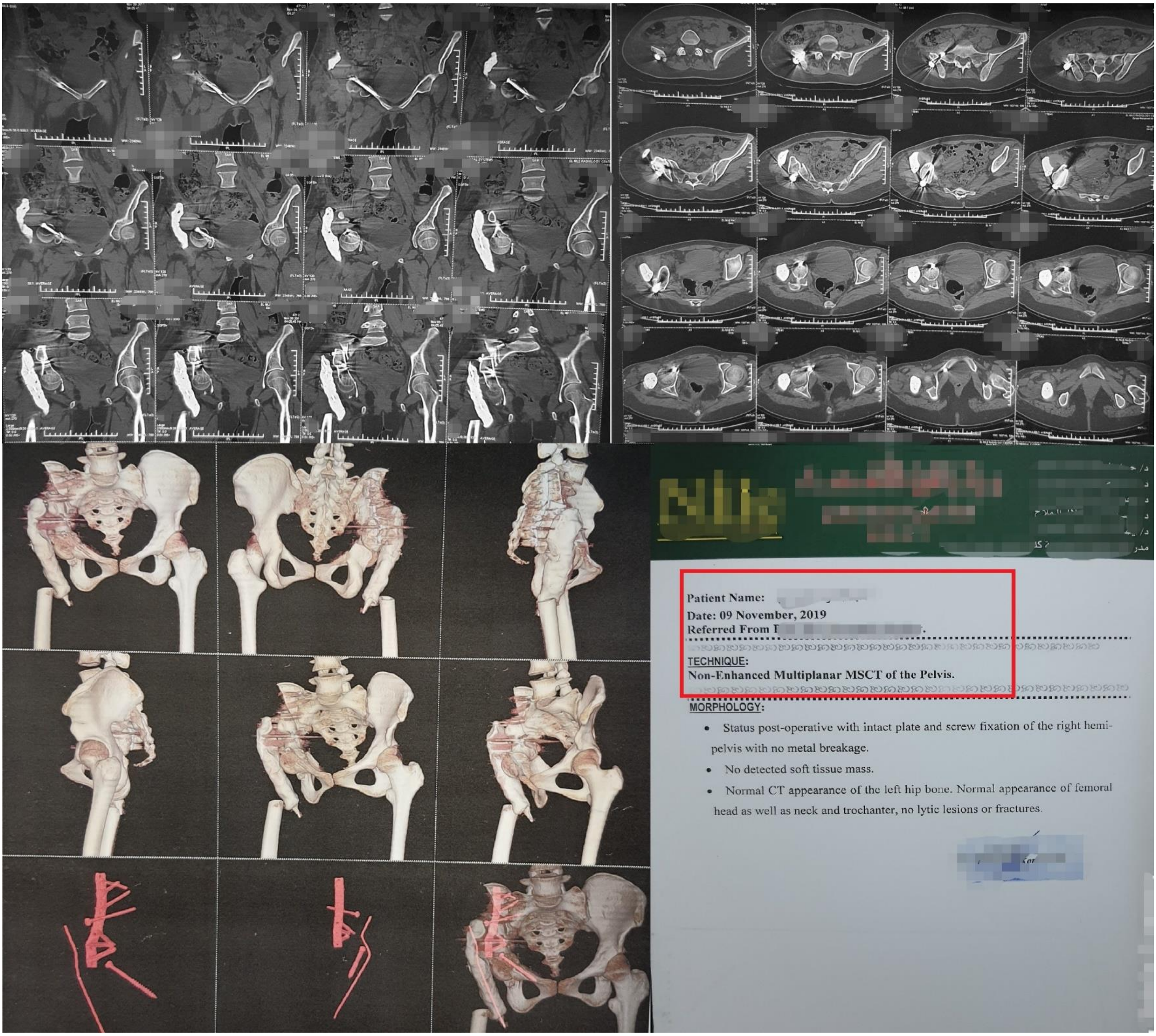

Figure S4: Microscopic examination images and report of the resected mass during the first surgical stage.

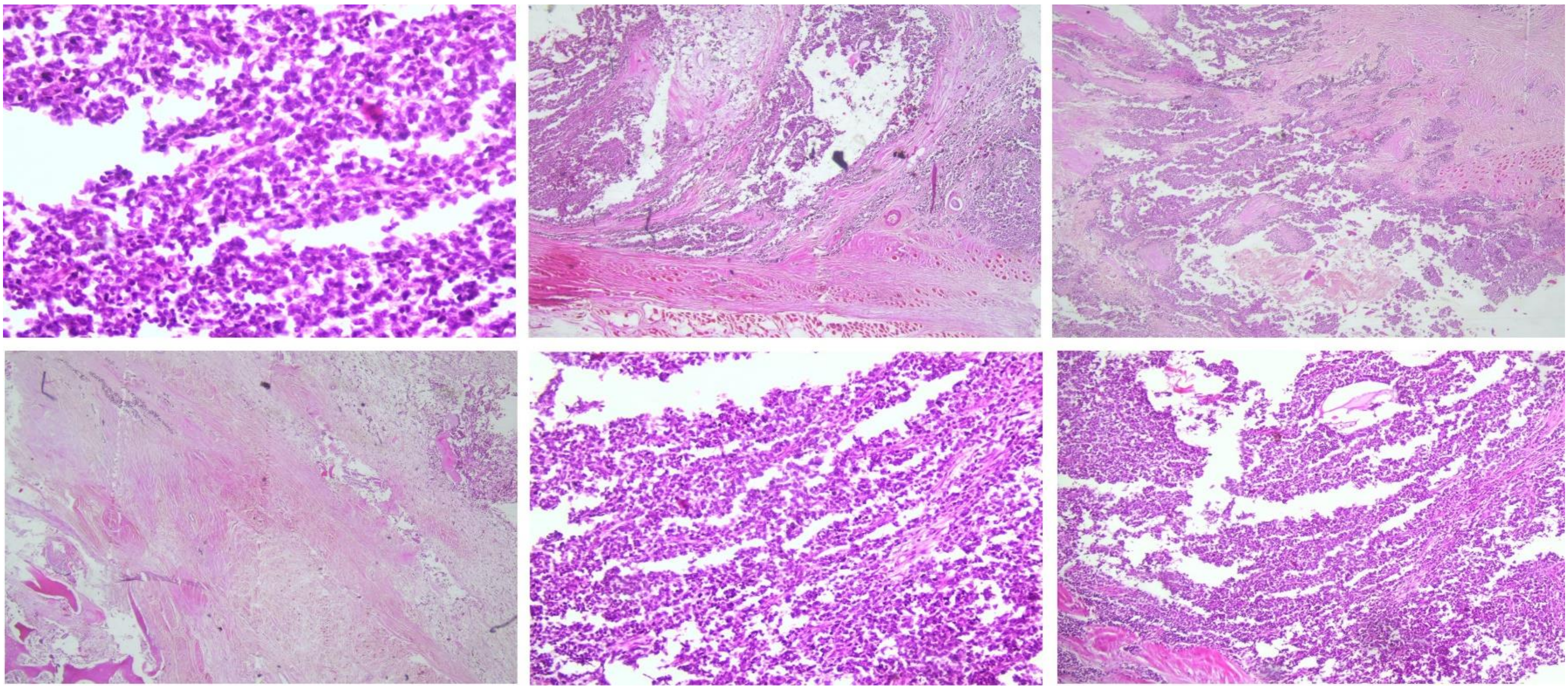

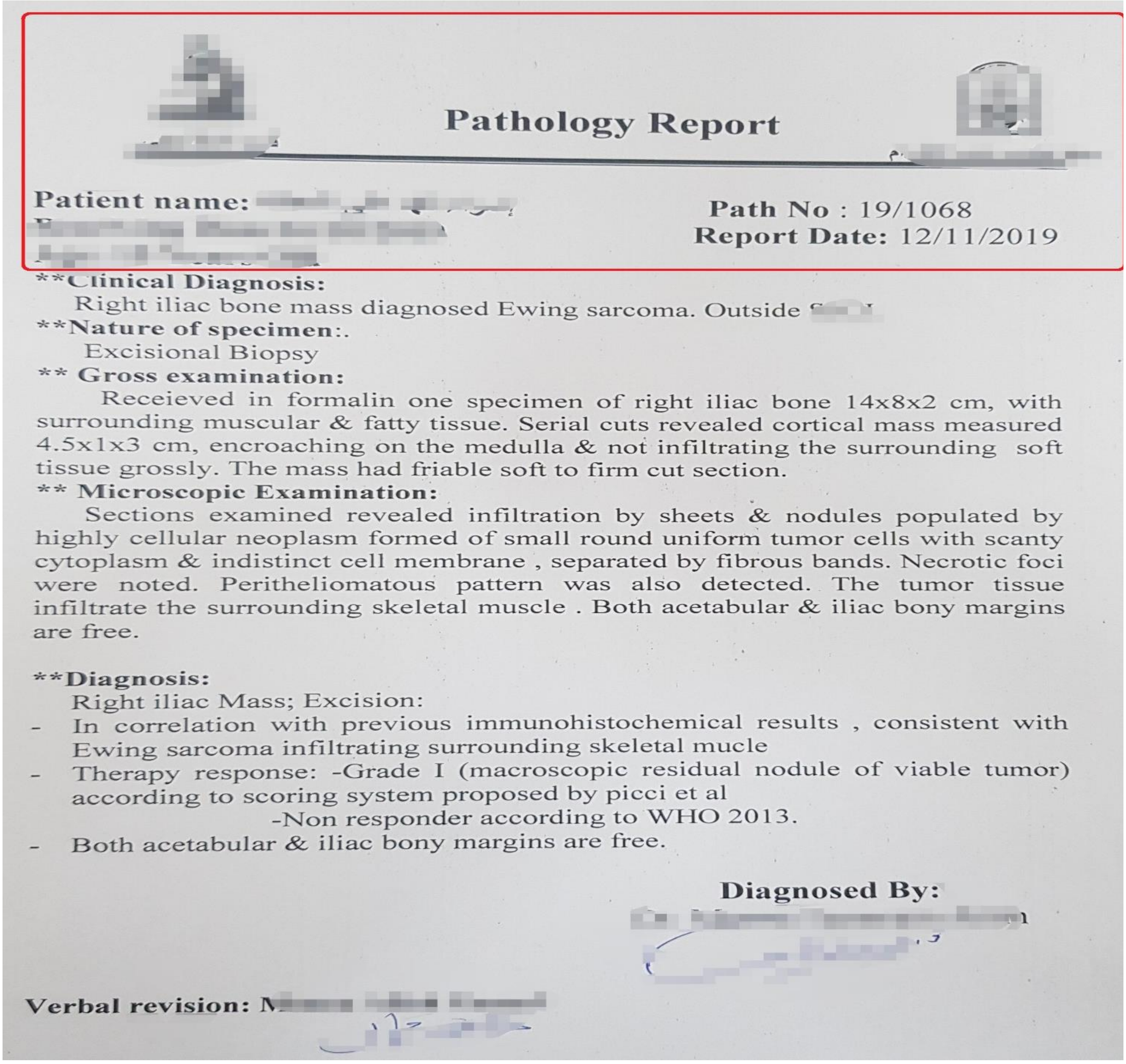

Pathology Report

Patient name: [illegible] **Path No** : 19/1068
[illegible] **Report Date:** 12/11/2019
[illegible]

****Clinical Diagnosis:**
Right iliac bone mass diagnosed Ewing sarcoma. Outside [illegible]

****Nature of specimen**:.
Excisional Biopsy

**** Gross examination:**
Receieved in formalin one specimen of right iliac bone 14x8x2 cm, with surrounding muscular & fatty tissue. Serial cuts revealed cortical mass measured 4.5x1x3 cm, encroaching on the medulla & not infiltrating the surrounding soft tissue grossly. The mass had friable soft to firm cut section.

**** Microscopic Examination:**
Sections examined revealed infiltration by sheets & nodules populated by highly cellular neoplasm formed of small round uniform tumor cells with scanty cytoplasm & indistinct cell membrane , separated by fibrous bands. Necrotic foci were noted. Peritheliomatous pattern was also detected. The tumor tissue infiltrate the surrounding skeletal muscle . Both acetabular & iliac bony margins are free.

****Diagnosis:**
Right iliac Mass; Excision:

- In correlation with previous immunohistochemical results , consistent with Ewing sarcoma infiltrating surrounding skeletal mucle
- Therapy response: -Grade I (macroscopic residual nodule of viable tumor) according to scoring system proposed by picci et al
  -Non responder according to WHO 2013.
- Both acetabular & iliac bony margins are free.

Diagnosed By:
[illegible]

Verbal revision: [illegible]
